# Natriuretic peptide receptor a promotes gastric malignancy through angiogenesis process

**DOI:** 10.1038/s41419-021-04266-7

**Published:** 2021-10-20

**Authors:** Zheng Li, Hao Fan, Jiacheng Cao, Guangli Sun, Sen Wang, Jialun Lv, Zhe Xuan, Yiwen Xia, Linjun Wang, Diancai Zhang, Hao Xu, Zekuan Xu

**Affiliations:** 1 Department of General Surgery, The First Affiliated Hospital of Nanjing Medical University, No.300, Guangzhou Road, Nanjing, 210029 Jiangsu Province China; 2 Jiangsu Key Lab of Cancer Biomarkers, Prevention and Treatment, Collaborative Innovation Center for Cancer Personalized Medicine, Nanjing Medical University, Nanjing, 210029 Jiangsu Province China

**Keywords:** Gastric cancer, Gastric cancer

## Abstract

Gastric cancer (GC) ranks the third among global cancer-related mortality, especially in East Asia. Angiogenesis plays an important role in promoting tumor progression, and clinical trials have demonstrated that anti-angiogenesis therapy is effective in GC management. Natriuretic peptide receptor A (NPRA) functions significantly in promoting GC development and progression. Whether NPRA can promote angiogenesis of GC remains unclear. Tumor samples collection and immunohistochemical experiment showed that the expression of NPRA was positively correlated with the expression of CD31 and vessel density. In vivo and in vitro analysis showed that NPRA could promote GC-associated angiogenesis and tumor metastasis. Results of Co-IP/MS showed that NPRA could prevent HIF-1α from being degraded by binding to HIF-1α. Protection of HIF-1α improved VEGF levels and thus promoted angiogenesis. In summary, NPRA protected HIF-1α from proteolysis by binding to HIF-1α, increased the expression of HIF-1α, and promoted GC angiogenesis. This study has discovered a new mechanism for NPRA to promote gastric cancer development and a new regulatory mechanism for HIF-1α.

## Introduction

Gastric cancer (GC) ranks third in the global cancer-related morbidity and mortality, especially in East Asia [1]. At present, the comprehensive treatment level of gastric cancer is constantly improving, but the overall survival (OS) rate is still low, especially for advanced gastric cancer, which is accompanied by recurrence and metastasis [2]. Novel strategies to suppress gastric cancer growth and metastasis should be developed. Angiogenesis has been well illustrated as one of the hallmarks of cancer, and is fundamental to tumor growth and metastasis [3, 4]. Tumor cells release pro-angiogenic cytokines and growth factors that form blood vessels and help them flourish [5]. The important role of angiogenesis in breast cancer, pancreatic cancer, gastric cancer, and other tumors has been extensively studied [6–8]. Inhibition of the angiogenesis signaling pathway will help prevent tumor growth and prolong the survival time of cancer patients [9, 10]. For gastric cancer, the REGARD trial, RAINBOW trial, and several ongoing RCTs have shown that ramucirumab that targeting VEGF signals help GC patients to achieve better survival [11, 12]. Despite the exciting results, the therapeutic targets for the angiogenesis process are not yet sufficient and remain to be discovered to prevent tumor-associated angiogenesis.

Angiogenesis is activated when pro-angiogenic signaling has the upper hand. Among the identified signaling pathways and molecules, vascular endothelial growth factor (VEGF), especially for VEGF-A is essential to activate the tumor angiogenesis process [5, 13]. VEGF holds the pro-angiogenic role by binding to its receptors, such as VEGFRA and VEGFRB. VEGF signaling is also regulated by several upstream molecules. Hypoxia-inducible factor-1 (HIF-1) is an oxygen-sensitive transcription factor that is upregulated in tumors hypoxic microenvironment [14, 15]. HIF-1 is easily degraded in an environment with sufficient oxygen. Hypoxia or protection by other proteins can increase the expression of HIF-1 [16–18]. Several studies have shown that HIF-1, especially HIF-1α promotes cancer growth and metastasis by activating angiogenesis in solid tumors containing GC [19–21]. However, the regulating mechanism of HIF-1 and VEGF remains unclear in GC and the discovery of the mechanisms will provide a potential therapeutic target.

Natriuretic peptide receptor A (NPRA) is the most important receptor of atrial natriuretic peptide (ANP), a well-illustrated natriuretic hormone that regulates body fluid volume and blood pressure [22]. NPRA not only conducts the biological effect of ANP, but also exerts its effect in tumorigenesis and progression [23, 24]. Our previous studies have shown that the expression of NPRA positively correlated to the tumor size and pathological stages of gastric cancer. NPRA inhibition resulted in impaired gastric cancer cell proliferation and viability both in vitro and in vivo. NPRA knockdown exerted its anti-tumor effect by impairing the mitochondria function and increasing the level of reactive oxygen species (ROS) [25]. However, the physiological role of NPRA is not completely clear and we wonder whether NPRA could promote GC development by activating angiogenesis. Considering that NPRA is a transmembrane polymer receptor protein and recycles in the cell, it may exert its physiological effects by binding and regulating other proteins [26, 27].

In this study, we found that NPRA could promote angiogenesis, which was identified by a series of in vivo and in vitro experiments in GC. We found that tumors with higher NPRA expression would have higher CD31 expression and greater vessel density. Higher NPRA expression predicted worse survival in GC patients. Tube formation assay and HUVEC transwell assay showed that NPRA could help GC cells secret VEGF and promote angiogenesis. Live imaging of small animals and mouse xenograft models showed that NPRA promoted vessel formation and tumor cell metastasis. Co-IP/MS showed that NPRA bound to HIF-1α and prevented HIF-1α from being degraded. More HIF-1α under NPRA protection would activate VEGF and angiogenesis. NPRA would be an important pro-angiogenic factor and therapeutic target of gastric cancer.

## Materials and methods

### Cell culture

All human GC cell lines MKN45, AGS, and NCI-N87 were purchased from Shanghai Institutes for Biological Sciences and human umbilical vein endothelial cells (HUVECs) were purchased from American type culture collection. All cells were cultured in RPMI 1640 (Gibco, USA) with 10% fetal bovine serum (FBS, Gibco, USA) and incubated in humidified chamber supplemented with 5% CO^2^ at 37˚C.

### Reagents and antibodies

Cycloheximide (CHX, C7698) and MG132 (HY-13259) were purchased from Sigma (USA) and MedChemexpress (USA). Anti-CD31 (ab28364) and anti-VEGF (ab32152) antibodies were purchased from Abcam (USA). Anti-HIF-1α (3434) antibodies were purchased from Cell Signaling Technology (USA). Anti-NPRA (sc-137041), anti-β-actin (sc-8432), anti-mouse IgG-HRP (sc-2005), and anti-rabbit IgG-HRP (sc-2004) antibodies were purchased from Santa Cruz (USA).

### Quantitative real-time reverse transcription polymerase chain reaction (qRT-PCR)

Total RNA extraction and quantitative real-time PCR (qRT-PCR) were performed as described previously [25]. The following primers were used in this study: HIF-1α forward, 5’-TCACCACAGGACAGTACAGGATGC-3’, HIF-1α reverse, 5’-CCAGCAAAGTTAAAGCATCAGGTTC-3’; β-actin forward, 5′-GCATCGTCACCAACTGGGAC-3′, β-actin reverse, 5′-ACCTGG CCGTCAGGCAGCTC-3′. β-actin was used as an endogenous control for mRNA.

### Lentivirus transfection and establishment of stably transfected cell line

shRNA sequences including shNPRA-1: 5’- GCATTCTGATTGTCTCCTTCT-3’, shNPRA-2: 5’-GGGTTGTACTGAACTACAATG-3’ and a nonsense sequence shCTL: 5’-GTTCTCCGAACGTGTCACGT-3’ packaged in lentivirus vectors were designed and synthesized by GenePharma (Shanghai, China). The HIF-1α overexpression plasmid was synthesized into the GV362 vector (Genechem, China). Transfections were performed using lipofectamie 2000 (Invitrogen, USA) following the manufacturer’s instructions.

### Western blot analysis and immunoprecipitation

Total protein was extracted using a protein extraction kit (Beyotime, China). Concentration-appropriate gels by SDS-PAGE were used for protein analysis and transferred onto a nitrocellulose membrane. Then, the membranes were blocked with 5% bovine serum albumin (BSA), incubated with specific primary antibodies at 4 °C overnight. After being washed with TBST at room temperature, the membranes were incubated with secondary antibodies for 2 h at room temperature, washed 3 times for 15 min each in TBST. Chemiluminescence HRP Substrate (Millipore, USA) were used to evaluate the expression level.

The protein A/G-agarose beads (Santa Cruz Biotechnology, USA) was used for the immunoprecipitation of cell lysates following the manufacturer’s instructions. Then, a western blot was used to detect the immunoprecipitated proteins.

### Immunoprecipitation and mass spectrometry (IP/MS)

Total proteins were extracted from GC cells and IP was performed using the indicated primary antibody and protein A/G-agarose beads (Santa Cruz Biotechnology, USA). The isolated immunoprecipitates were then analyzed by MS (Thermo Scientific, USA).

### Tube formation assay

Matrigel (BD Biosciences, USA) was thawed at 4 °C overnight, then added to 96-well plate for 50 μL/well, and coated at 37 °C for 1 h. HUVECs were seeded into the 96-well plate with 5000 cells per well. Images were taken after 8 h, and Image-Pro Plus (IPP) software was used to measure and calculate tube area.

### Cell counting kit-8 assay (CCK-8)

CCK-8 (Dojindo, Japan) was used for cell proliferation assay. 2000 HUVECs were seeded into each well in 96-well plates and incubated for 3 days. The standard microplate reader (Scientific MultiskanMK3, Thermo Scientific) was used to record the absorbance at 450 nm every 24 h according to the manufacturers’ protocols.

### Cell migration and invasion assays

A Transwell system with a 6.5 mm diameter chamber (Corning, USA) were employed to assess the cell migration and invasion ability. HUVECs were pre-cultured in different treated conditioned mediums. For migration assays, HUVECs treated differently were cultured with a non-FBS medium in the upper chamber, whereas a medium containing 10% FBS was used in the lower chamber. After 72 h incubation, the cells in the upper chamber were then removed using cotton swabs. For invasion assays, cells were seeded on matrigel-coated membrane inserts. The following process are as the migration assays. Then, crystal violet was used to stain the cells that migrating or invading into the bottom of the membrane of the inserts. Then inserts were washed with PBS for 2 times and counted using an inverted microscope.

### Cell cycle arrest and apoptosis measurement by flow cytometry

HUVECs treated differently were harvested and carefully washed with PBS. Then, 75% ethanol was used to fix the cells and stored at −20 °C overnight. Afterwards, cell cycle distribution was detected by cell cycle detection kit (Multisciences, China). The Annexin V-APC/PI Apoptosis Detection Kit (Multisciences, China) was used to stain the apoptotic cells according to the manufacturer’s instructions. Cell cycle arrest and apoptosis were analyzed by flow cytometry after reagent pretreatment.

### Human organoid culture

The human organoid culture was performed as our previously reported [28]. Sterile gastric cancer tissues were cut into small pieces and digested with collagenase A. Then, the Matrigel (R&D Systems, USA) supplemented with growth factors was used to resuspend cells. After that, Organoid Growth Medium (human) (StemCell Technologies, Canada) was used to culture the mixtures that were seeded in a 24-well plate. Photographs of human GC organoids were taken daily by microscope.

### ELISA assay

VEGF, mainly refers to as VEGFA, is secreted protein, so we employed ELISA assay to test the VEGFA level in different conditioned mediums. The control and NPRA-knockdown cells were seeded in 6-well plates (1.5 × 10^5^ per well), and incubated for 24 h. Then the culture supernatant was collected and the cell number was calculated. The supernatant was used for the ELISA test as manufacturer’s instructions (R&D systems, USA).

### Mouse xenograft model establishment

4-week-old female nude mice were purchased and raised in the Department of Laboratory Animal Centre of Nanjing Medical University (Nanjing, China). Pathogen-free conditions were guaranteed. The number of 10^6^ cells treated differently were injected into the flanks of the mice (6 mice each group). The tumor volumes were measured every five days and calculated with the following formula: tumor volume = (length × width^2^)/2. After 3 weeks, mice were sacrificed and the xenograft tumors were harvested. The metastasis model was constructed by injecting cells into the caudal vein of anesthetized nude mice (3 mice each group) and monitored using IVIS imaging systems (Caliper Life Sciences, USA). All experimental procedures were in compliance with the guidelines of the First Affiliated Hospital of Nanjing Medical University (NMU).

### Immunohistochemical (IHC), Hematoxylin and eosin (HE) staining and Tissue microarray (TMA) analysis

86 tumor samples were collected from GC patients who received operations in the First Affiliated Hospital of Nanjing Medical University. Tissue microarray (TMA) was constructed and immunohistochemistry staining were done according to a previous study [25]. CD31 and NPRA were stained to analyze the vessel density and NPRA expression in GC tissues or xenograft tumors. The immunoreactive score (IRS) was used in expression assessment. The staining intensity was graded as 0, 1, 2, and 3 (no staining, weak, moderate and strong, respectively). The proportion of positive cells was scored as 0, 1, and 2 (<10%, 10–50%, and >50%, respectively). Following formula combined the two scores: IHC score = positive rate score × intensity score. Paraffin-embedded lung sections of mice were stained by HE and cut into 5 μm slices for pathological evaluation and observation under a microscope (Olympus, Japan). Written informed consent was obtained from each patient. This study was approved by the First Affiliated Hospital of NMU.

### Statistical analysis

Experimental data were shown as mean ± standard deviation (SD). Means were calculated from at least three independently performed experiments. GraphPad Prism 7.0 (GraphPad Software, USA) and SPSS software ver. 20.0 (IBM, USA) were used for data analyses. Differences between groups were evaluated using by the two-tailed student t-test, analysis of variance (ANOVA) or χ2 test. *P*-values <0.05 was considered as statistically significant.

## Results

### NPRA expression was positively associated with the vessel density and the tumor stage

**Fig. 1:**
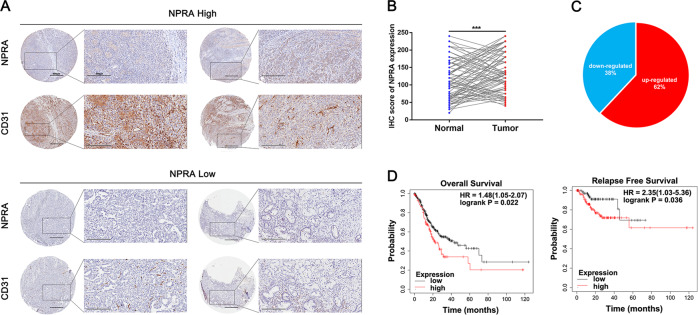
NPRA was upregulated in GC and associated with poor prognosis. **A** The expression of NPRA and CD31 from GC tissues were measured by IHC. **B**, **C** The protein level of NPRA was significantly increased in GC tissues (53/86). **D** Online Kaplan–Meier overall survival (OS) and relapse-free survival (RFS) curves according to NPRA expression levels. ***p < 0.001.

In order to evaluate the pro-angiogenic role of NPRA, we employed a tissue microarray which was constructed of 86 tumor tissues. The TMA was stained with the NPRA and CD31 specific antibodies to assess the NPRA expression and vessel density, and the results indicated that the NPRA protein level was significantly increased in 62% (53/86) of GC tissues, higher NPRA expression would have higher CD31 expression (Fig. 1A–C). Western blot analysis also showed that higher NPRA expression was indeed accompanied by increased expression of CD31 in tumors (Fig. S1), which was consistent with the result of immunostainings. Clinical and pathological information was acquired and used in the following analysis. As shown in supplementary table 1, higher NPRA expression tented to possess the larger tumor size and more advanced tumor stages, which was consistent with our previous study. In addition, in tumor tissues with higher NPRA expression, the expression of CD31 was significantly increased (Table S1). We conducted the survival analysis of the TCGA cohort with the help of KMPLOT (http://kmplot.com/analysis/). The results suggested that GC patients with higher NPRA expression had the worse overall survival (OS) and relapse-free survival (RFS) (Fig. 1D). These findings indicated that NPRA was upregulated in gastric cancer and may be related to angiogenesis in gastric cancer.

### NPRA increased angiogenesis and promoted VEGF signaling pathway

**Fig. 2:**
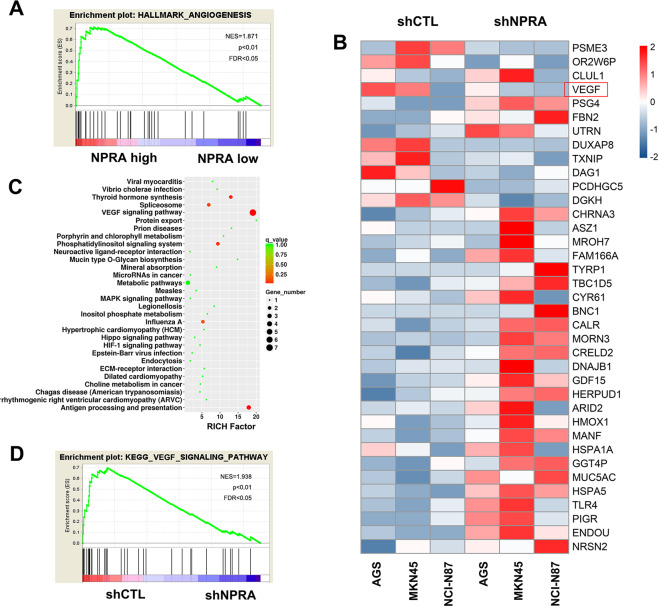
NPRA promoted angiogenesis by regulating VEGF signaling pathway in GC. **A** GSEA analysis of the relation of NPRA expression with angiogenesis based on TCGA cohort. **B** The cluster heat map was used to analyze gene expression levels of subsets of genes in shNPRA GC cells relative to matched shCTL GC cells. **C** KEGG pathway analysis of the RNA-seq results from shNPRA and the corresponding shCTL GC cells. **D** GSEA analysis of subsets of genes related to the VEGF signaling pathway.

By the TMA construction and following immunohistochemical tests, we found that the expression of NPRA was positively correlated with the expression of CD31, the vessel marker. As shown in Fig. 1A, higher NPRA expression indicated higher CD31 expression and vessel density in the same location of the slides. In order to illustrate the regulating patterns of NPRA on the angiogenesis signaling, we performed gene set enrichment analysis (GSEA) based on the TCGA dataset to further explore its possible mechanism underlying GC, the results implied that NPRA expression was positively associated with angiogenesis (Fig. 2A). Next, we employed the next generation sequencing (NGS) towards three pairs of control and NPRA-knockdown GC cell lines and analyzed the signaling pathways regulated by NPRA via a KEGG analysis. As shown in Fig. 2B, VEGF was among the most downregulated genes after NPRA knockdown. VEGF signaling pathways were also affected after the inhibition of NPRA (Fig. 2C-D). These results indicated that NPRA may accelerate angiogenesis by promoting VEGF signaling in GC.

### NPRA promoted GC-associated angiogenesis in vitro

**Fig. 3:**
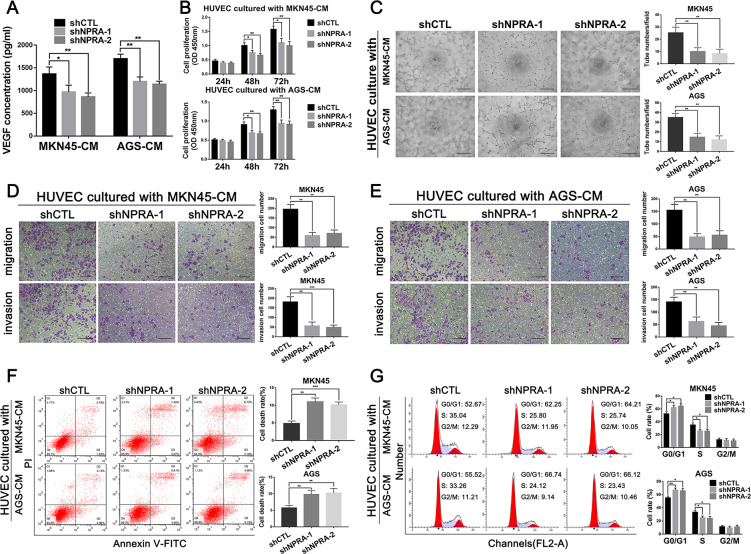
NPRA promoted GC-associated angiogenesis in vitro. **A** The expression of VEGF in the culture medium of MKN45 and AGS cells transfected with shNPRA and the control group (shCTL) was detected by ELISA assay. **B** CCK8 assay was applied to detect cell viability of HUVECs cultured with different conditioned mediums of shNPRA and shCTL GC cells. **C** Tube-formation assays with HUVECs were performed with the different groups of GC conditioned medium, scale bar: 100 μm. **D**, **E** Transwell migration and invasion assays with HUVECs treated with different conditioned mediums showed that knockdown of NPRA reduced the cell ability of migration invasion, scale bar: 200 μm. **F**, **G** Flow cytometry assay was used to analyze the HUVEC cell viability and cell cycle regulated by different GC cells conditioned mediums. CM: conditioned medium, *p < 0.05, **p < 0.01, ***p < 0.001.

Through tumor sample testing and NGS analysis, we have found that NPRA could promote GC-associated angiogenesis. We next worked to explore whether NPRA could promote angiogenesis in vitro. As VEGF is a “trigger” in promoting angiogenesis, we tested the concentration of VEGF in conditioned medium of MKN45-shCTL, MKN45-shNPRA, AGS-shCTL, and AGS-shNPRA cells. As shown in Fig. 3A, the VEGF level was obviously downregulated in NPRA-knockdown GC cells. Meanwhile, we investigated the influence of NPRA on the proliferation, tube formation, migration, invasion, cell cycle, and apoptosis of HUVEC cells using the above GC conditioned medium. As shown in Fig. 3B, by CCK-8 proliferation assay, we found that knockdown of NPRA could dramatically attenuate the HUVEC growth in GC-conditioned medium compared with the negative control (shCTL). NPRA silencing also induced impaired tube formation capacities in tube formation assay (Fig. 3C). HUVEC migration and invasion abilities are important in promoting angiogenesis. Knockdown of NPRA could impair HUVEC migration and invasion ability compared with the negative control (shCTL) (Fig. 3D-E). Flow cytometry assay was performed to analyze the HUVEC cell cycle and cell viability regulated by GC cells conditioned medium. As shown in Fig. 3F, the viability of HUVEC treated with a conditioned medium of NPRA inhibited GC cells were significantly impaired. Cell cycle analysis showed that HUVEC co-cultured with NPRA knockdown GC cells conditioned medium manifested an obvious cell cycle arrest in the G0/G1 phase and the number of cells in the S phase was reduced dramatically, which meant impaired proliferation (Fig. 3G).

**Fig. 4:**
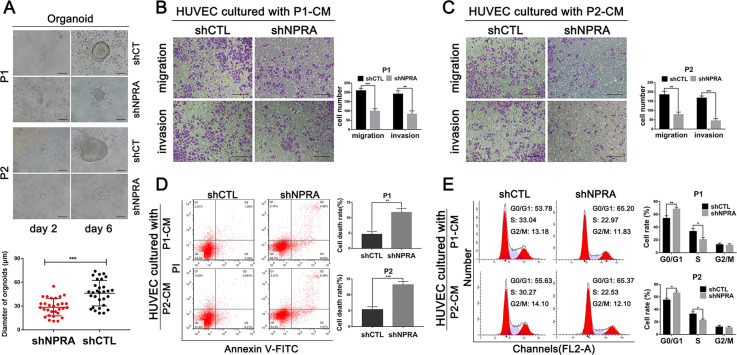
Silencing NPRA could inhibit growth of the GC organoids. **A** Effects of NPRA knockdown on the growth of gastric organoids, scale bar: 20 μm. **B**, **C** Transwell migration and invasion assays with HUVECs treated with conditioned medium of Patiente1-shCTL, Patiente1-shNPRA, Patiente2-shCTL, and Patiente2-shNPRA GC organoids, scale bar: 200 μm. **D**, **E** Flow cytometry assay was used to analyze the HUVEC cell viability and cell cycle that regulated by different GC organoids conditioned medium. CM: conditioned medium, *p < 0.05, **p < 0.01, ***p < 0.001.

In addition, the GC organoid models were further employed to investigate the role of NPRA in tumor growth. We discovered that the knockdown of NPRA could inhibit the growth of the GC organoids (Fig. 4A). Then we continued to use the conditioned medium of Patient1-shCTL, Patient1-shNPRA, Patient2-shCTL, and Patient2-shNPRA GC organoids to culture HUVECs. We found that VEGF level was obviously downregulated in NPRA-knockdown GC organoids conditioned medium (Fig. S2A). Transwell migration and invasion assays and flow cytometry assays were performed to evaluate the effects of NPRA on cell proliferation, cycle, and apoptosis of HUVECs, the results were consistent with what we found in GC cells conditioned medium above (Fig. 4B-E). These results indicated that NPRA may promote GC-associated angiogenesis in vitro.

### NPRA facilitated GC-associated angiogenesis in vivo

**Fig. 5:**
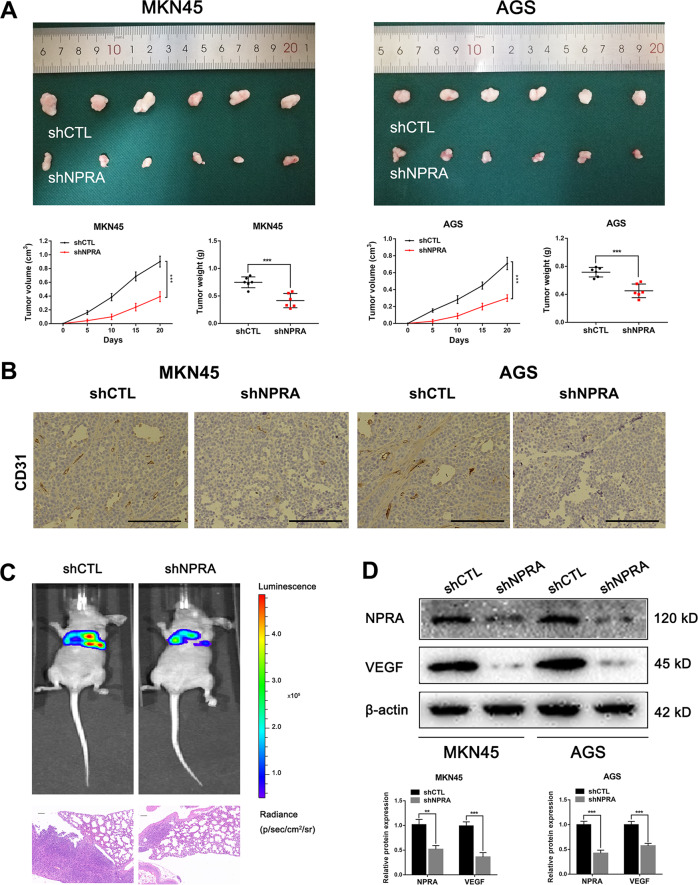
NPRA facilitated GC-associated angiogenesis in vivo. **A** Xenograft tumors in the nude mouse model was treated with shNPRA and shCTL. **B** CD31 expression of NPRA-silencing xenografted tumors were significantly lower than control groups, scale bar: 50 μm. **C** Representative images of lung metastasis and HE staining of the specimen, scale bar: 100 μm. **D** The VEGF detection of tumor harvested from mouse xenograft model by western blot. **p < 0.01, ***p < 0.001.

In order to detect the angiogenesis-promoting ability in vivo, we employed the mouse xenograft model. Tumor cells were injected subcutaneously into the flanks of the mice. As shown in Fig. 5A, NPRA silencing cells formed smaller tumors as what we found in our former studies. Xenografted tumors were harvested and analyzed with immunohistochemical staining of CD31 to mark the vessels. CD31 expression of NPRA silencing xenografted tumors were significantly lower than control groups (Fig. 5B). Next, stable transfected AGS cells treated differently were injected into the tail vein of BALB/c nude mice separately to explore the function of NPRA on tumor metastasis in vivo. The results indicated that knockdown of NPRA alleviated lung metastasis (Fig. 5C). The VEGF detection of tumor harvested from the mouse xenograft model by western blot showed that the expression of VEGF was impaired when NPRA was inhibited (Fig. 5D). All these results suggested that knockdown of NPRA could inhibit angiogenesis, tumor growth, and metastasis in vivo in GC.

### NPRA positively regulated HIF-1α expression resulting angiogenesis promotion

**Fig. 6:**
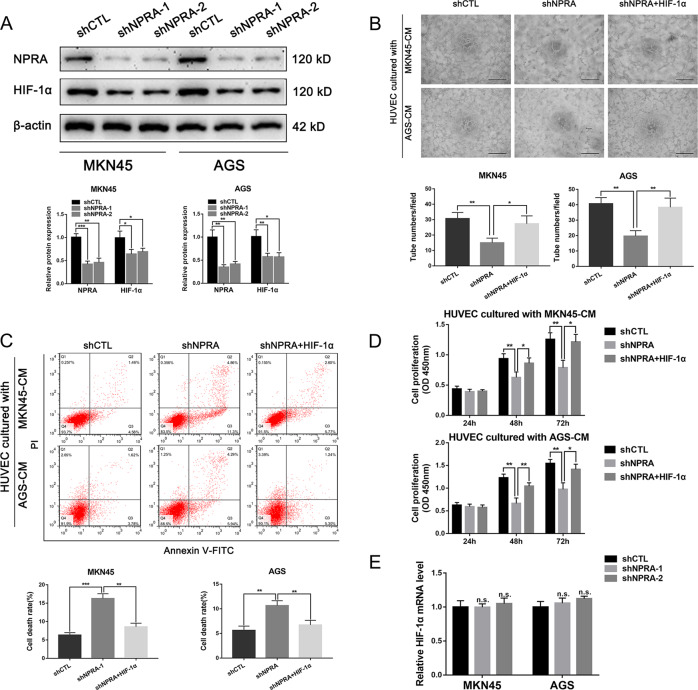
NPRA positively regulated HIF-1α expression resulting in angiogenesis promotion. **A** Western blots were employed to assess the HIF-1α expression regulated by NPRA. **B** Tube formation assays of HUVECs treated with the GC conditioned medium of shCTL, shNPRA, and shNPRA+HIF-1α, respectively, scale bar: 100 μm. **C**, **D** Flow cytometry assay and CCK8 assay were used to analyze the HUVEC cell viability and cell proliferation that regulated by the above GC conditioned medium. **E** qRT-PCR detection showed that NPRA had no significant influences on HIF-1α expression on mRNA level. CM: conditioned medium, *p < 0.05, **p < 0.01, ***p < 0.001.

HIF-1α is an important angiogenesis factor in tumors. We next worked to evaluate whether NPRA could regulate HIF-1α in the angiogenesis process. Western blot was performed to assess the correlation between NPRA and HIF-1α expression. Our results showed that the expression of NPRA was positively correlated with the expression of HIF-1α (Fig. 6A). Then we cultured HUVECs with a conditioned medium of MKN45-shCTL, MKN45-shNPRA, MKN45-shNPRA + HIF-1α, AGS-shCTL, AGS-shNPRA, and AGS-shNPRA + HIF-1α cells, and VEGF concentration was detected (Fig. S2B). Tube formation assays showed that overexpression of HIF-1α in NPRA-inhibiting cells could rescue the angiogenesis impairment of shNPRA cells (Fig. 6B). CCK8 assay and Flow cytometry assay also showed that overexpression of HIF-1α could reverse HUVEC proliferation and viability impaired by knockdown of NPRA (Fig. 6C-D). We have also used the transwell experiment to explore the rescue function of HIF-1α to NPRA, and we found that overexpression of HIF-1α could rescue HUVEC migration and invasion ability impaired by knockdown of NPRA (Fig. S3). qRT-PCR detection showed that NPRA had no significant influences on HIF-1α expression on mRNA level (Fig. 6E). These results indicated that NPRA may promote GC-associated angiogenesis through post-translationally regulating the important angiogenesis factor HIF-1α.

### NPRA promoted tumor angiogenesis by binding and protecting HIF-1α against protein degradation

**Fig. 7:**
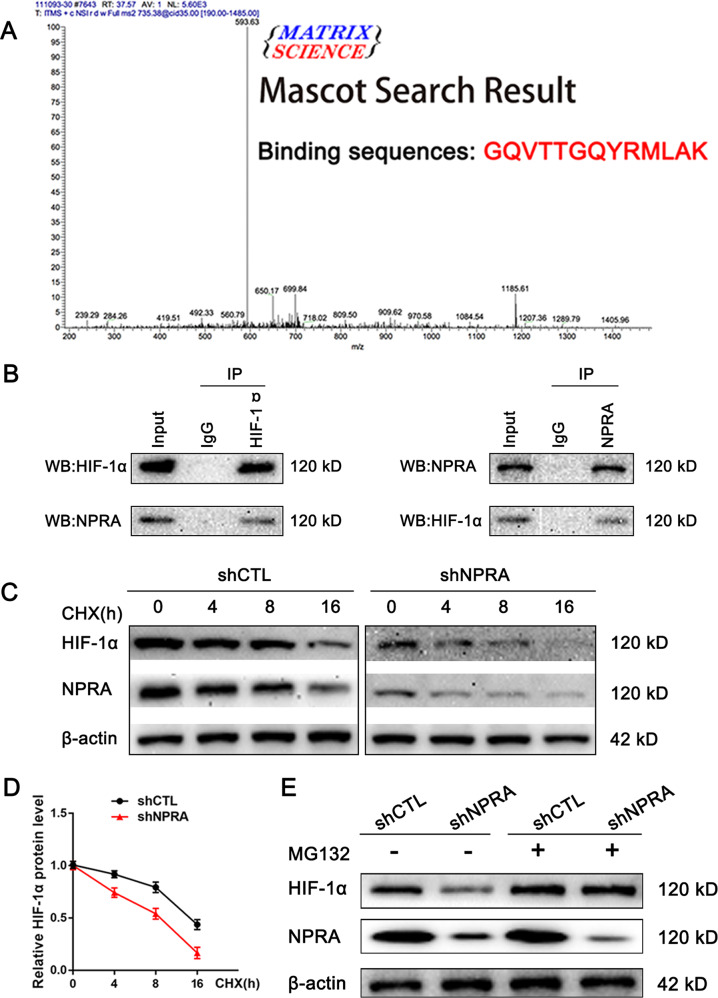
NPRA promoted tumor angiogenesis by binding and protecting HIF-1α against protein degradation. **A** The proteins that bound to NPRA were determined by immunoprecipitation/mass spectrum (IP/MS) analysis, which indicated that NPRA could bind to HIF-1α at high binding scores. **B** Co-IP experiments demonstrated that NPRA immunoprecipitated with anti- HIF-1α antibody, and HIF-1α immunoprecipitated with anti- NPRA antibody. **C**, **D** Western blot analysis of HIF-1α protein stability using cycloheximide (CHX, an inhibitor of protein synthesis) when knockdown of NPRA. **E** AGS cells transfected with shNPRA and shCTL were treated with MG132, HIF-1α, and NPRA protein levels were detected by western blot.

**Fig. 8:**
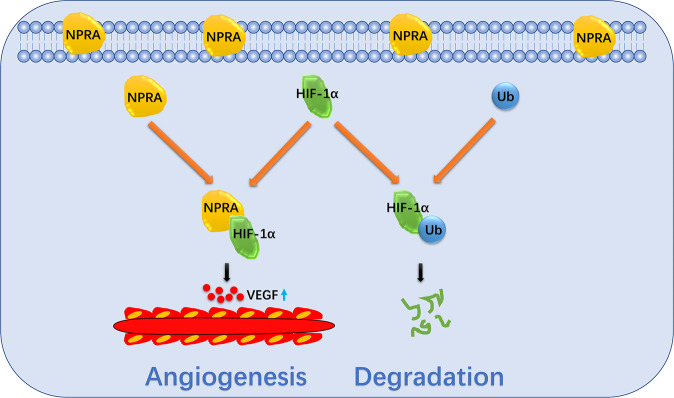
Schematic representation of the role of NPRA on the angiogenesis in GC. In summary, NPRA promoted GC angiogenesis by activating the HIF-1α–VEGF axis. NPRA interacted with HIF-1α protein and stabilized it to prevent its ubiquitination degradation.

As we mentioned before, HIF-1α is an unstable protein that is easily ubiquitinated and degraded. Studies have reported that some polymer proteins can protect HIF-1α from degradation by binding to HIF-1α, thereby promoting its expression and pro-angiogenesis function [17, 18]. NPRA is a high-molecular protein in the cell membrane and cytoplasm. Whether the angiogenesis ability of NPRA is related to the binding of HIF-1α remained to be uncovered. Co-immunoprecipitation assays were conducted to see the binding proteins of NPRA. As shown in Fig. 7A-B, the mass spectrometry suggested NPRA could bind to HIF-1α at high binding scores, the anti-NPRA antibody specifically co-immunoprecipitated HIF-1α and the anti-HIF-1α antibody specifically co-immunoprecipitated NPRA, revealing that NPRA interacts with HIF-1α in AGS cells. We then wondered whether NPRA affected the stability of HIF-1α. The protein stability assay of HIF-1α using cycloheximide (CHX, an inhibitor of protein synthesis) revealed that HIF-1α was decreased when knockdown of NPRA (Fig. 7C-D). Then we incubated AGS cells with MG-132 to reverse the protein degradation. Results suggested that after the MG-132 supplement, the expression of HIF-1α in NPRA-silencing GC cells recovered significantly (Fig. 7E). These results suggested that by combining HIF-1α, NPRA protected the HIF-1α from ubiquitination and degradation, increased the expression of HIF-1α, and improved the angiogenesis abilities of gastric cancer (Fig. 8).

## Discussion

Gastric cancer is among the most leading causes of cancer-related death, especially in China [1]. So far, there is no effective treatment towards advanced and metastatic GC. Traditional chemotherapy and targeted therapy have made progress in GC treatment, while drug resistance would develop not in a long time. Angiogenesis is fundamental for tumor development, inhibiting angiogenesis signals has been turned out to be effective in GC treatment [29]. Clinical trials such as RAINBOW and REGARD trials have shown that anti-VEGF ramucirumab could prolong the survival of advanced GC patients alone or with traditional chemotherapy [11, 12]. Laboratory studies have also found that angiogenesis could promote the development of gastric cancer [30, 31]. Interventions on the key proteins of angiogenesis signaling can effectively suppress the proliferation and activity of gastric cancer cells.

In this study, we identified that NPRA, our previously reported oncogene that promoted gastric cancer by eliminating ROS accumulation that threatens tumors [25], could promote GC-associated angiogenesis and tumor metastasis in vitro and in vivo. Inhibition of NPRA is accompanied by a reduced VEGF concentration. Our further work found that HIF-1α, a key factor that promotes angiogenesis, can bind to NPRA, and NPRA can elevate HIF-1α levels. HIF-1 is one of the most important key factors in regulating GC-associated angiogenesis [32, 33]. HIF-1 has been identified as a heterodimer composed of a subunit HIF-1α and a subunit HIF-1β [34]. HIF-1α is the indispensable factor in promoting angiogenesis [35]. HIF-1α is prone to proteolysis in an environment with sufficient oxygen, so the high expression of HIF-1 can be maintained in a relatively hypoxic environment in tumor tissues. The expression of HIF-1α and its regulated genes can be detected in relative hypoxic regions, such as 100 μm from effectual blood vessels in tumor tissues [36]. The expression of HIF-1α induced by a hypoxic environment can promote GC-related angiogenesis, increase the blood supply in the tumor tissues, and thus continuously supply the rapid growth of the tumor. However, the increase in blood vessel density can bring more oxygen to tumor tissues, threatening the stability of HIF-1α. Under hypoxia, P402 and P564 residues in the oxygen-dependent degradation domain of HIF-1α are hydroxylated by prolyl-4-hydroxylases (PHDs) [37, 38]. Prolyl-hydroxylation attracted the pVHL-containing E3 ubiquitin ligase and triggered the proteolysis of HIF-1α through the ubiquitin-proteasome system [39–41].

In reality, the pro-angiogenesis process of tumor cells will never stop, but will continue with the growth of the tumor. Therefore, there must be other mechanisms to protect HIF-1α from being degraded, thereby continuously promoting gastric cancer angiogenesis and tumor development. Studies have shown that HIF-1α can bind to other proteins by which HIF-1α could escape from been degradation. Lee et al. showed that BICD1 could bind to and protect HIF-1α which enhanced the regenerative potential of mesenchymal stem cells [42]. It reminded us that discovery of partners of HIF-1α was of great significance for regulating the HIF-1α signaling and angiogenesis process.

NPRA is a guanylate cyclase-combined membrane receptor, which mediates the majority of physiological effects of ANP [22]. Our previous study revealed that NPRA could accelerate cell proliferation and activity by reducing the level of ROS in gastric cancer cells, and promote the development of gastric cancer [25]. As a high-molecular-weight transmembrane protein, NPRA is rapidly internalized, sequestrated, and redistributed into intracellular locations after binging the ligand [43]. Thus, NPRA is thought to be a cellular protein that travels around different subcellular locations. This physiological characteristic determines that it may play its physiological role as a chaperone protein of other molecules in the cytoplasm. This study confirmed that NPRA could bind to HIF-1α and maintain the stability of HIF-1α, which provided a basis for NPRA to promote angiogenesis of gastric cancer. Our research data showed that inhibiting the expression of NPRA could lead to the impaired HIF-1α protein level, and inhibiting the proteasome could rescue the HIF-1α expression. The co-IP assay has also identified the combination of HIF-1α and NPRA. This study found a new regulation mechanism of HIF-1α, and NPRA as a membrane protein may have the possibility of becoming a target of drug therapy. Inhibitory treatment for NPRA may further block tumor angiogenesis, thereby reducing tumor nutrition supply and inhibiting tumor growth.

In summary, NPRA protects HIF-1α from proteolysis by binding to it and increases the expression of HIF-1α which promotes the angiogenesis of GC. This study has discovered a new mechanism for NPRA to promote gastric cancer development and a new regulatory mechanism for HIF-1α. This study can be used as the basis for inhibiting angiogenesis in gastric cancer by inhibiting NPRA.

## Supplementary information

supplementary table 1

figure S1

figure S2

figure S3

supplementary figure legends

cddis-author-contribution-form

## Data Availability

The data that support the findings of this study are available from the corresponding author upon reasonable request.
