# Supplementary material for: Natriuretic peptide receptor a promotes gastric malignancy through angiogenesis process
Source: Cell Death Dis. 2021 Oct 20;12(11):968. doi: 10.1038/s41419-021-04266-7 (PMC8528824; doi:10.1038/s41419-021-04266-7)
Supplement: Supplementary file 1 — supplementary table 1 [file 41419_2021_4266_MOESM1_ESM.docx]

| Characteristics | Number | NPRA expression | | **P-value** |
| --- | --- | --- | --- | --- |
|  |  | **High** | **Low** |  |
| Age(years) |  |  |  |  |
| <60 | 34 | 19 | 15 | 0.376 |
| ≥60 | 52 | 34 | 18 |  |
| **Gender** |  |  |  |  |
| Male | 60 | 38 | 22 | 0.621 |
| Female | 26 | 15 | 11 |  |
| **Size** |  |  |  |  |
| <3 | 34 | 11 | 23 | **< 0.001** |
| ≥3 | 52 | 42 | 10 |  |
| **Differentiation** |  |  |  |  |
| Well + Moderate | 35 | 18 | 17 | 0.107 |
| Poor + signet | 51 | 35 | 16 |  |
| **Lymphatic metastasis** |  |  |  |  |
| Yes | 39 | 25 | 14 | 0.667 |
| No | 47 | 28 | 19 |  |
| **Invasion depth** |  |  |  |  |
| T1+T2 | 41 | 23 | 18 | 0.314 |
| T3+T4 | 45 | 30 | 15 |  |
| **TNM stage** |  |  |  |  |
| I+II | 40 | 16 | 24 | **< 0.001** |
| III+IV | 46 | 37 | 9 |  |
| **CD31** |  |  |  |  |
| High | 49 | 37 | 12 | **0.002** |
| Low | 37 | 16 | 21 |  |

Supplementary table 1. Correlation between NPRA expression and clinicopathological characteristics of gastric cancer patients.

P<0.05 was considered significant
