# Supplementary figures and images for: Natriuretic peptide receptor a promotes gastric malignancy through angiogenesis process
Source: Cell Death Dis. 2021 Oct 20;12(11):968. doi: 10.1038/s41419-021-04266-7 (PMC8528824; doi:10.1038/s41419-021-04266-7)

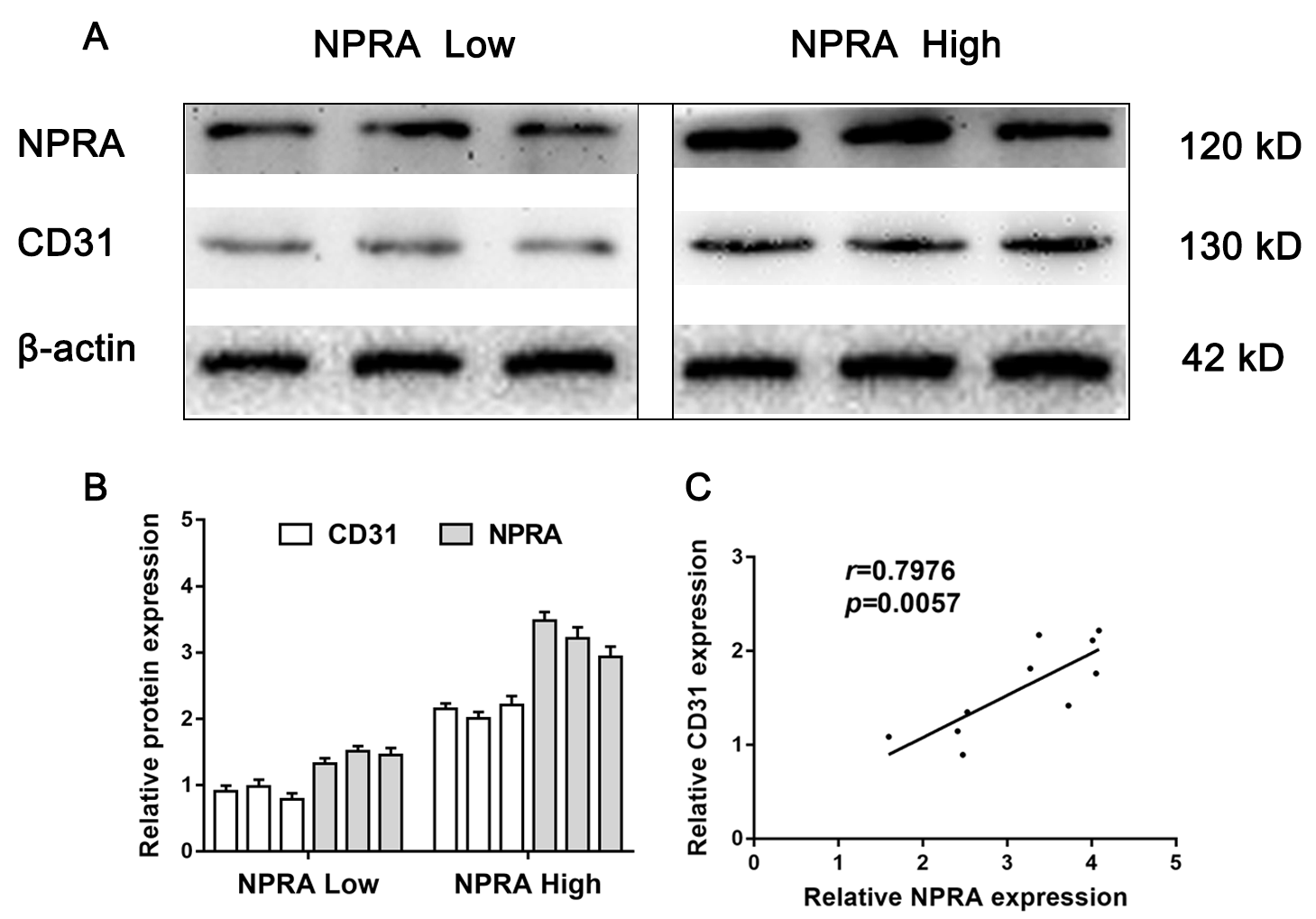

Supplement: Supplementary file 2 — figure S1 [file 41419_2021_4266_MOESM2_ESM.tif]

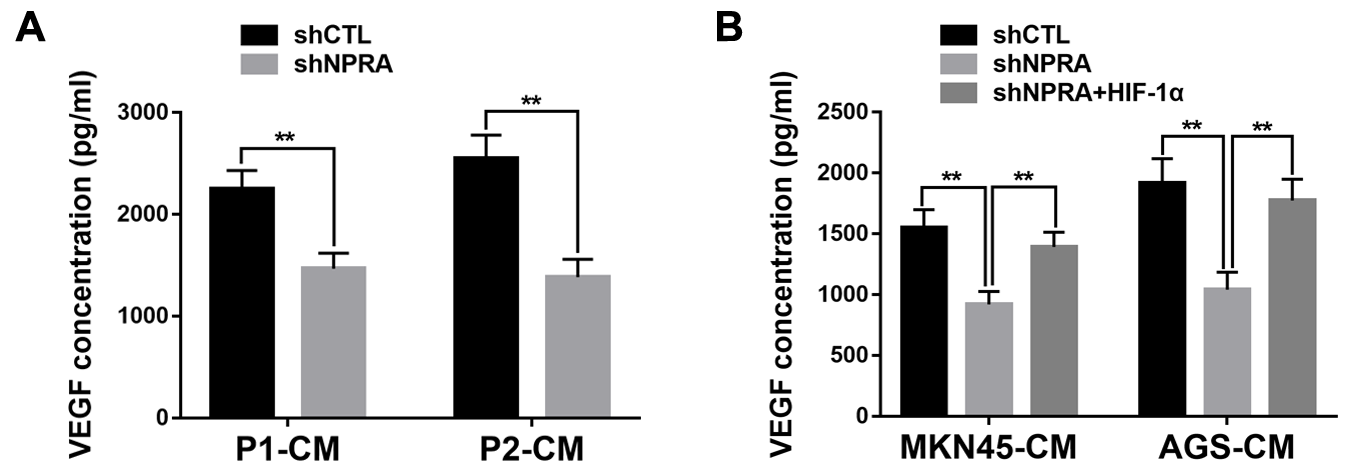

Supplement: Supplementary file 3 — figure S2 [file 41419_2021_4266_MOESM3_ESM.tif]

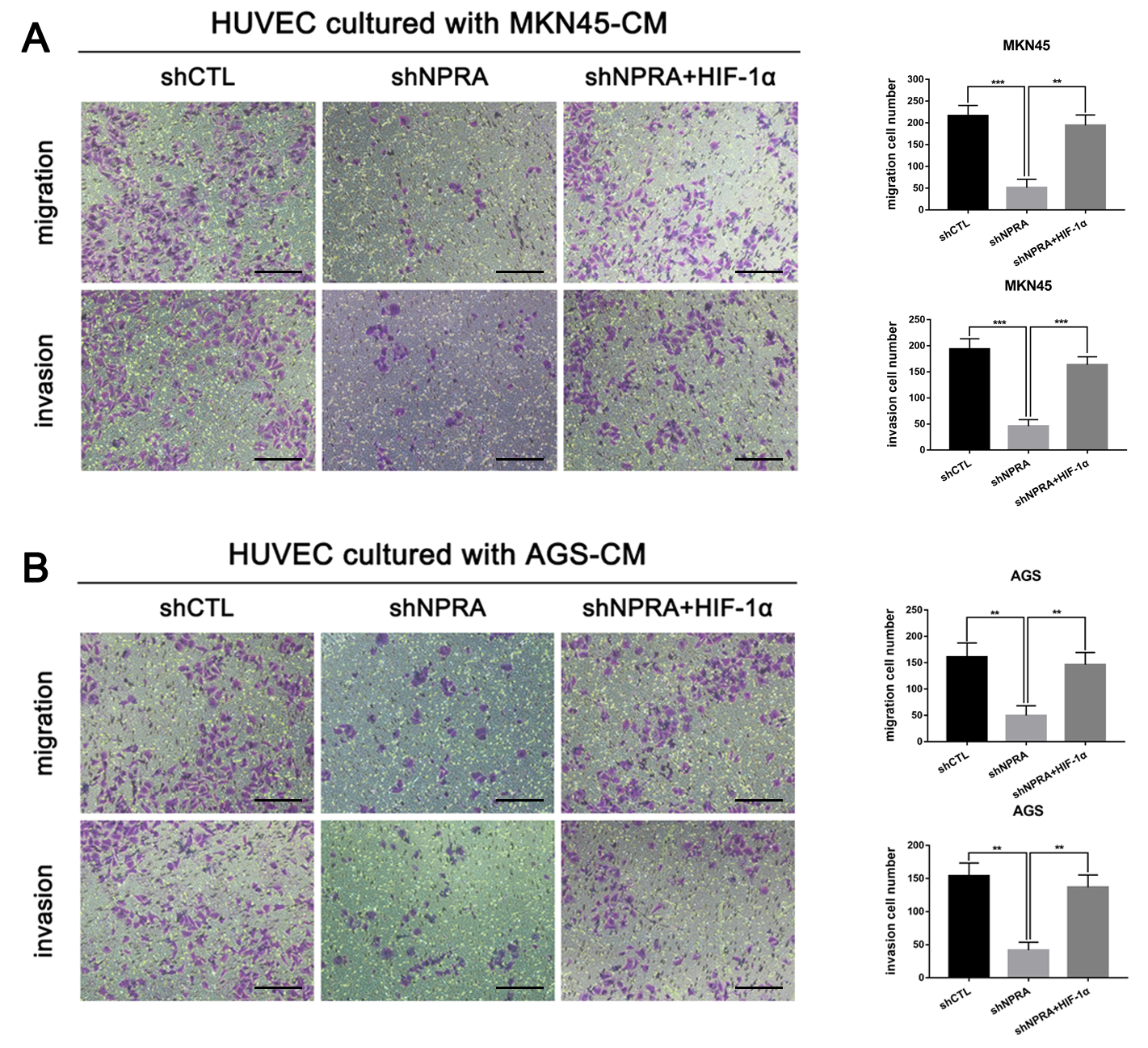

Supplement: Supplementary file 4 — figure S3 [file 41419_2021_4266_MOESM4_ESM.tif]
