# Supplementary material for: Natriuretic peptide receptor a promotes gastric malignancy through angiogenesis process
Source: Cell Death Dis. 2021 Oct 20;12(11):968. doi: 10.1038/s41419-021-04266-7 (PMC8528824; doi:10.1038/s41419-021-04266-7)
Supplement: Supplementary file 5 — supplementary figure legends [file 41419_2021_4266_MOESM5_ESM.docx]

**Natriuretic peptide receptor A promotes gastric malignancy through** **angiogenesis process**

Zheng Li^1^, Hao Fan^1^, Jiacheng Cao^1^, Guangli Sun^1^, Sen Wang^1^, Jialun Lv^1^, Zhe Xuan^1^, Yiwen Xia^1^, Linjun Wang^1^, Diancai Zhang^1^, Hao Xu^1^, Zekuan Xu^1,2^

^1^Department of General Surgery, The First Affiliated Hospital of Nanjing Medical University, No.300, Guangzhou Road, Nanjing 210029, Jiangsu Province, China.

^2^Jiangsu Key Lab of Cancer Biomarkers, Prevention and Treatment, Collaborative Innovation Center for Cancer Personalized Medicine, Nanjing Medical University, Nanjing 210029, Jiangsu Province, China.

**Supplementary Figure Legends**

Supplementary Figure 1.

A,B. Protein expression from GC tissues showed that higher NPRA expression accompanied by increased expression of CD31. C. Correlation analysis of the expression of NPRA and CD31 in 10 GC tissues.

Supplementary Figure 2.

A. The expression of VEGF in the conditioned medium of Patient1-shCTL, Patient1-shNPRA, Patient2-shCTL and Patient2-shNPRA GC organoids was detected by ELISA assay. B. The expression of VEGF in the culture medium of MKN45 and AGS cells transfected with shCTL, shNPRA and shNPRA+ HIF-1α was detected by ELISA assay. CM: conditioned medium, **p < 0.01.

Supplementary Figure 3.

A,B. Transwell assays were used to detect the cell migration and invasion ability after culturing HUVECs with the conditioned medium of MKN45-shCTL, MKN45-shNPRA, MKN45-shNPRA + HIF-1α, AGS-shCTL, AGS-shNPRA and AGS-shNPRA + HIF-1α cells. CM: conditioned medium, **p < 0.01, ***p < 0.001.
