# Supplementary material for: Natriuretic peptide receptor a promotes gastric malignancy through angiogenesis process
Source: Cell Death Dis. 2021 Oct 20;12(11):968. doi: 10.1038/s41419-021-04266-7 (PMC8528824; doi:10.1038/s41419-021-04266-7)
Supplement: Supplementary file 6 — cddis-author-contribution-form [file 41419_2021_4266_MOESM6_ESM.pdf]

DECLARATION OF CONTRIBUTIONS TO ARTICLE

ADMC

Manuscript Number:	Journal Name:	
CDDIS-21-1965	*Cell Death & Disease*	(the 'Journal')

Proposed Title of the Contribution:

Attribution of Authorship (the 'Contribution)

Author(s):

Zheng Li, Hao Fan, Jiacheng Cao, Guangli Sun, Sen Wang, Jialun Lv, Zhe Xuan, Yiwen Xia, Linjun Wang, Diancai Zhang, Hao Xu, Zekuan Xu (the 'Authors')

For all *CDDis* articles, each person named as an author in the published version must be able to show he or she has contributed substantially to the article.

Authorship credit should be based on 1) substantial contributions to conception and design, acquisition of data, or analysis and interpretation of data; 2) drafting the article or revising it critically for important intellectual content; and 3) final approval of the version to be published. Authors should meet conditions 1, 2 and 3.

Any person who cannot be shown to have made a substantial contribution to the article cannot be listed as an author in the final version. The name of any person who is deemed to have made a minor contribution can, however, appear in the Acknowledgments section of the article.

Please complete the table below to indicate the contributions of all named authors to the manuscript.

Author Full Name:	Specification of Contribution to the Manuscript:
Zheng Li	acquisition of data,drafting and revising the article, analysis and interpretation of data
Hao Fan	acquisition of data,drafting and revising the article, analysis and interpretation of data
Jiacheng Cao	analysis and interpretation of data, drafting and revising the article, analysis and interpretation of data
Guangli Sun	analysis and interpretation of data, drafting and revising the article, analysis and interpretation of data
Sen Wang	analysis and interpretation of data
Jialun Lv	analysis and interpretation of data
Zhe Xuan	acquisition of data
Yiwen Xia	acquisition of data
Linjun Wang	analysis and interpretation of data, revising the article
Diancai Zhang	analysis and interpretation of data, revising the article
Hao Xu	analysis and interpretation of data, revising the article
Zekuan Xu	conception and design the study, revising it critically for important intellectual content

Please complete the table below to indicate the contributions of all named authors to the figures.

Figure 1:

Sen Wang acquired the data, Zheng Li analyzed and interpreted the data, Zheng Li assembled the figure.

Figure 2:

Jialun Lv acquired the data, Hao Fan analyzed and interpreted the data, Hao Fan assembled the figure.

Figure 3:

Zhe Xuan acquired the data, Jiacheng Cao analyzed and interpreted the data, Jiacheng Cao assembled the figure.

Figure 4:

Yiwen Xia acquired the data, Guangli Sun analyzed and interpreted the data, Guangli Sun assembled the figure.

Figure 5:

Linjun Wang and Diancai Zhang acquired the data, Zekuan Xu analyzed and interpreted the data, Zheng Li assembled the figure.

Figure 6:

Hao Xu acquired the data, Zekuan Xu and Zheng Li analyzed and interpreted the data, Zheng Li assembled the figure.

Signed for and on behalf of the Author(s):	Print Name:	Date:
	Zekuan Xu	2021-05-21
